# Pleiotropic Outcomes of Glyphosate Exposure: From Organ Damage to Effects on Inflammation, Cancer, Reproduction and Development

**DOI:** 10.3390/ijms222212606

**Published:** 2021-11-22

**Authors:** Marianna Marino, Elena Mele, Andrea Viggiano, Stefania Lucia Nori, Rosaria Meccariello, Antonietta Santoro

**Affiliations:** 1 Dipartimento di Medicina, Chirurgia e Odontoiatria “Scuola Medica Salernitana”, Università degli Studi di Salerno, Via S. Allende, 84081 Baronissi, Italy; mamarino@unisa.it (M.M.); aviggiano@unisa.it (A.V.); 2 Dipartimento di Scienze Motorie e del Benessere, Università degli Studi di Napoli Parthenope, 80133 Naples, Italy; elena.mele@collaboratore.uniparthenope.it; 3 Dipartimento di Farmacia, Università degli Studi di Salerno, Via Giovanni Paolo II, 84084 Fisciano, Italy; snori@unisa.it

**Keywords:** glyphosate toxicity, inflammation, cancer, reproduction and development

## Abstract

Glyphosate is widely used worldwide as a potent herbicide. Due to its ubiquitous use, it is detectable in air, water and foodstuffs and can accumulate in human biological fluids and tissues representing a severe human health risk. In plants, glyphosate acts as an inhibitor of the shikimate pathway, which is absent in vertebrates. Due to this, international scientific authorities have long-considered glyphosate as a compound that has no or weak toxicity in humans. However, increasing evidence has highlighted the toxicity of glyphosate and its formulations in animals and human cells and tissues. Thus, despite the extension of the authorization of the use of glyphosate in Europe until 2022, several countries have begun to take precautionary measures to reduce its diffusion. Glyphosate has been detected in urine, blood and maternal milk and has been found to induce the generation of reactive oxygen species (ROS) and several cytotoxic and genotoxic effects in vitro and in animal models directly or indirectly through its metabolite, aminomethylphosphonic acid (AMPA). This review aims to summarize the more relevant findings on the biological effects and underlying molecular mechanisms of glyphosate, with a particular focus on glyphosate's potential to induce inflammation, DNA damage and alterations in gene expression profiles as well as adverse effects on reproduction and development.

## 1. Introduction

Glyphosate (N-phosponmetyl glycine; CAS registry number 1071-83-6; molecular formula C_3_-H_8_-N-O_5_-P) is one of the most widely used herbicides in the world. It was patented under the trade name of Roundup in 1974 by the Monsanto agrochemical industry. Monsanto’s patent expired in 2001 and since then glyphosate has been manufactured by many companies, including Bayer, a leading producer of glyphosate in Germany. Glyphosate and glyphosate-based herbicides (GBHs) were authorized for agricultural use in 1974 in the USA by the Environmental Protection Agency (EPA) while, in Europe, it was commercialized in 2002 after the approval of the European Commission [1].

Glyphosate, formulated as Roundup, is a broad-spectrum herbicide. Its main function is to eliminate weeds in crops of wheat, canola and soybeans, even though, in wheat crops, it is also used as a desiccant before the harvest [2]. In addition, glyphosate is used in urban areas, in parks and in marine contexts to eliminate aquatic plants [3]. In the mid-1990s the use of glyphosate and GBHs increased rapidly because of the development of glyphosate-resistant GM crops. In 2012, 127,000 tons of glyphosate were used in the USA and 700,000 tons worldwide [4]. Indeed, the USA is the nation where there is the largest use of glyphosate and GBHs. More than 750 products on the US market contain it and their use has been approved in more than 130 countries [5,6,7]. Due to its massive use, glyphosate is detectable in the air, water and in foodstuffs and consequently in biological fluids as urine, blood and maternal milk [8]. However, the specificity of glyphosate in inhibiting the activity of 3-phosphoshikimate 1-carboxyvinyltransferase (EPSPS), the key enzyme of the shikimate pathway, which is absent in vertebrates, has led, for a long time, to the idea that this pesticide could not represent a serious risk for human health [9]. While it is true that epidemiological studies on humans have reported only a possible, slight rise in the development of non-Hodgkin’s lymphomas among glyphosate-exposed farmers, in-vitro studies, however, have shown that this substance causes genetic damage, increases oxidative stress, interferes with the estrogen pathway, can be able to compromise brain functions and has been correlated with various forms of cancer [10,11,12]. With reference to the above evidence, the health effects of glyphosate have become a topic of crucial importance for research. The International Agency for Research on Cancer (IARC) carried out an in-depth analysis of research studies on the effects of glyphosate in humans and animals. This analysis ended, in 2015, with the decision to include this pesticide in the group 2A (probably carcinogenic to humans). In 2015, the European Food Safety Authority (EFSA) conducted another technical assessment entrusted to the German Federal Institute for Risk Assessment (BFR) and, from these valuations, it emerged that glyphosate was unlikely to represent a carcinogenic hazard for humans. Both views were met with criticism among the scientific community and society at large. Consequently, recent years have witnessed a plethora of studies on glyphosate toxicity and several reviews and opinion articles have also emerged in the peer-reviewed literature. In addition, the BFR is based in Germany, the country in which one of the main producers of glyphosate is based, and three consultants from the glyphosate agrochemical industry resided in the technical commission of the BFR. In November 2017 the UE decided to extend the authorization for glyphosate use until 2022. Although the judgment on the human health risk of glyphosate remains still uncertain, several countries have begun to take precautionary measures to reduce the inappropriate use of glyphosate and GBHs [5].

From the above observations, it emerges that the unsafety of glyphosate and GBHs represents a real emergency, therefore the aim of this review is to update the knowledge about the effects of this herbicides with particular reference to its immunomodulatory action, mutagenic/carcinogenic potential and its effects on reproduction and development. Through critically analyzing the overall aspects of glyphosate toxicity, we provide our insights, with aim to raise awareness about its use.

## 2. Glyphosate Action and Contamination Routes

**Figure 1:**
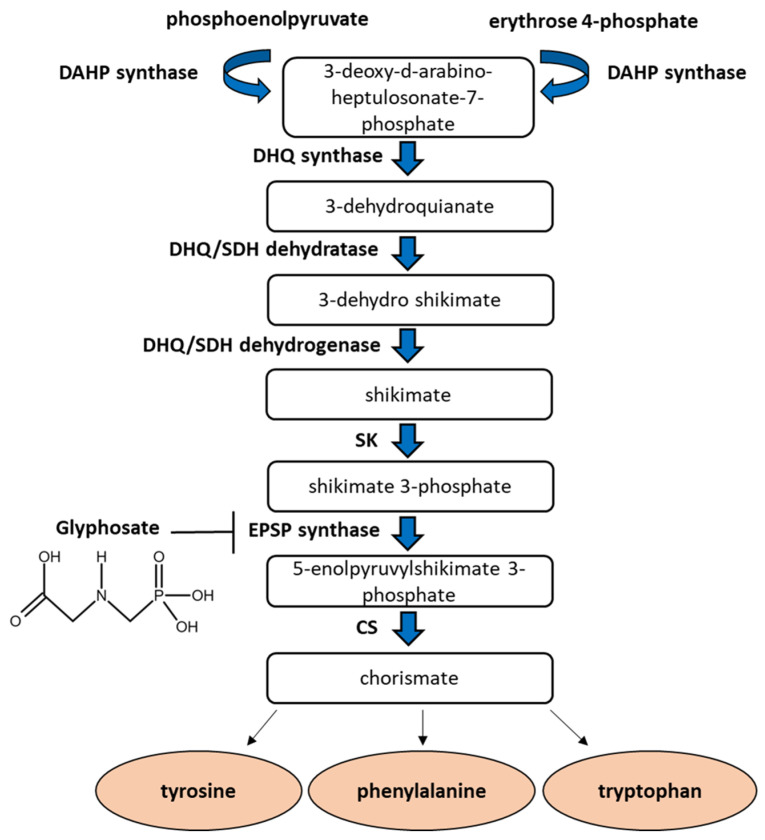
The shikimate pathway in plants. The shikimate pathway converts phosphoenolpyruvate (PEP) and erythrose 4-phosphate (E 4-P) into chorismate, the precursor of three aromatic amino acids. Glyphosate inhibits the 5-enolpyruvylshikimate 3-phosphate (EPSP) synthase enzyme, preventing this synthesis. DAHP: 3-deoxy-d-arabino-heptulosonate-7-phosphate synthase; DHQ: 3-deidroquianate synthase; DHQ/SDH dehydratase: 3-dehydroquianate dehydratase; DHQ/SDH dehydrogenase: 3-dehydroquianate dehydrogenase; SK: shikimate kinase; CS: chorismate synthase.

Glyphosate inhibits the activity of EPSPS, an enzyme required in the shikimate pathway for the synthesis of the aromatic amino acids alanine, tyrosine and phenylalananine in plants [13] (Figure 1). Aromatic amino acids are necessary to obtain different compounds that perform regulatory and defense functions, the lack of these amino acids lead to plant death [9,14].

**Figure 2:**
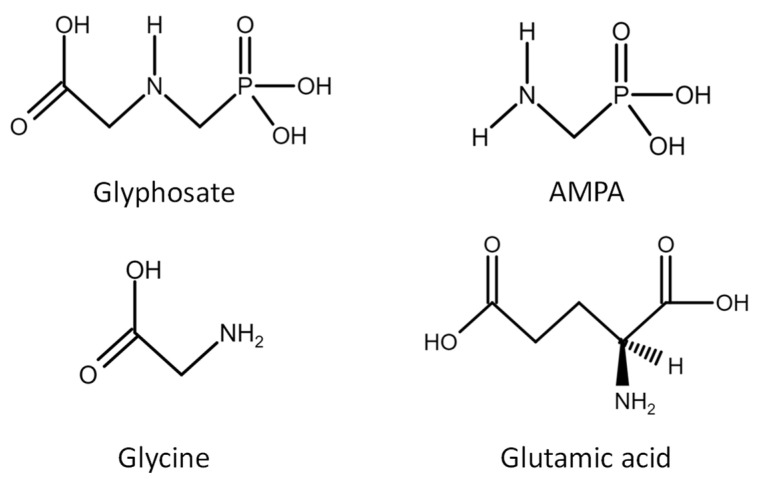
Chemical structures of glyphosate and its co-genres: aminomethylphosphonic acid (AMPA), glycine and glutamic acid.

The presence of the carbon phosphate bond (Figure 2) makes glyphosate quite resistant to degradation. Despite this, the degradation of glyphosate involves the breakdown of the carbon–nitrogen (C–N) bond, which leads to the production of aminomethylphosphonic acid (AMPA), the main metabolite of glyphosate [15,16]. However, glyphosate itself has a low toxicity and GBHs contain other substances that help glyphosate's entry into plants, increasing its toxicity. For example, it has been shown that Roundup is more toxic than glyphosate alone [17]. Roundup includes the co-formulant polyetholoxylated tallow amine (POEA) which appears to be the ingredient with the greatest toxic effects [18,19].

Furthermore, GBHs are applied to crops several times each season, both for removal of weeds and for drying before the grain harvest. The decomposition of glyphosate takes place in living plants as well as in the soil, this means that both glyphosate and its metabolites, such as AMPA, can be found in plant products [2,20].

The wide use of glyphosate and its ability to accumulate in the environment has increased concerns about the possible side effects that this compound can have on human health. GBHs tend, in fact, to accumulate in soil, water, plant products and food including grains, fruits and cereals, and they are not removed by washing nor degraded by cooking [21,22]. Recent research has shown that glyphosate is present in significant quantities in the meat of cattle and in the urine of the cows that consume contaminated food [23]. Indeed, there are not yet sufficient studies explaining the pharmacokinetics of glyphosate in vertebrates and its transport and bioaccumulation in various tissues. It has been shown that, in rats, after the oral ingestion of 400 mg/kg of pesticide, the concentration of glyphosate in the blood is equal to 5 μg/mL. In the 5 h following administration, glyphosate diffuses into the tissues, and it has half-lives, in terms of distribution and elimination, of 4 and 15 h, respectively [24].

In a study lead at the Ramazzini Institute, Sprague Dawley rats were subjected to oral administration, for 13 weeks, of water containing glyphosate or Roundup at levels equal to the United States Acceptable Daily Intake (US-ADI). It was found that glyphosate is excreted in the urine as a compound not modified and in greater quantities with respect to its main metabolite AMPA (see Figure 2 for the chemical structure). The amount of glyphosate present in urine increases in relation to the duration of treatment suggesting a possible bioaccumulation [16]. Accordingly, other in-vivo studies showed that once in the body glyphosate tends to accumulate in the kidneys, liver, colon and is then excreted via the feces or urine [12,16,25].

**Figure 3:**
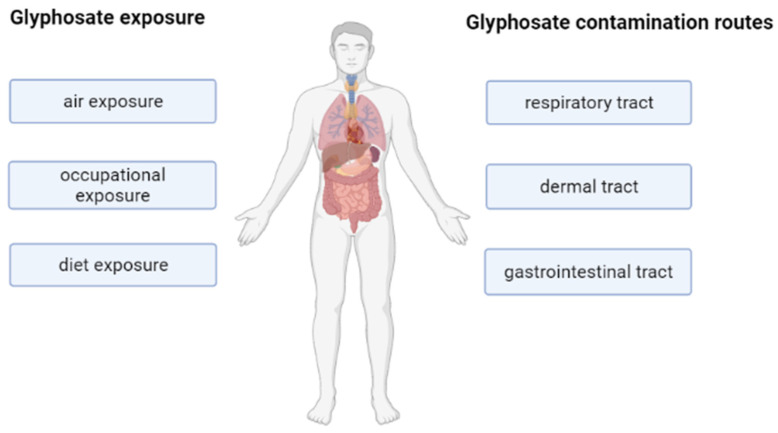
Scheme of the main exposure and contamination routes of glyphosate in humans. Image has been created with BioRender.com, accessed on 1 November 2021.

Although not a direct target, humans can be contaminated through occupational exposure and diet [21,26,27]. The preferential entry route is the dermal route. However, glyphosate can get into the human body also by inhalation, ingestion or by eating contaminated food [22] (Figure 3). On this hand, glyphosate has been found in high proportions in the urine of farmers and other biological fluids, such as blood and maternal milk, and it is also present in 60–80% of general population, including children [2,28]. This suggests that daily exposure to this pesticide, as well as its presence in the human body, could compromise human health.

Several recent reviews [2,13,14,29] have shown a significant correlation between the onset of various chronic diseases and the increased use of glyphosate, however, to date there are not sufficient data explaining how glyphosate and GBHs could alter human and animal metabolism and the molecular pathways targeted by glyphosate have not been completely elucidated. Available data suggest that the chemical structure of glyphosate and its metabolite AMPA are similar to those of glycine and glutamate (Figure 2), agonists of n-methyl-D-aspartate receptor (NMDAR) [30,31]. Moreover glyphosate, acting as an analogue of the amino acid glycine, can be mistakenly included in polypeptide chains during protein synthesis, generating abnormalities in proteins that play a fundamental role in metabolic and regulatory processes. The replacement of glyphosate with preserved glycine residues might explain, in part, the reason for the correlation between glyphosate exposure and the onset of various diseases [32]. In fact, recent findings obtained in MDA-MB-231 breast cancer cells did not show any difference between global proteome changes in glyphosate treated and untreated cells; only ADP/ATP translocase and serine/arginine—rich-splicing factor 6 exhibited statistically significant modifications in glyphosate treated cells [33], suggesting that the pesticide can target specific proteins. Indeed, glyphosate exposure seems to be involved in various diseases of modern era, such as obesity, diabetes, liver and kidney dysfunction, autism, dementia, Parkinson’s and Alzheimer’s disease, leukemia, various forms of cancer and inflammatory diseases.

## 3. Immunomodulatory and Inflammatory Effects of Glyphosate

### 3.1. Glyphosate—Induced Effects in Liver, Kidney and Lung

Many studies have found that glyphosate exerts its toxicity inducing inflammation and oxidative stress in various types of cells [34,35,36,37,38,39,40]. Oxidative stress, due to the excessive production of reactive oxygen species (ROS) or to poor antioxidants defenses, can damage proteins, lipids and activate apoptotic pathways and/or the onset of inflammatory processes. El-Shenawy et al. [35] demonstrated an oxidative stress response in Albino male rats intraperitoneally administered with sub-lethal concentrations of Roundup or glyphosate alone. They found a significant time-dependent depletion of GSH and induction of oxidative stress in hepatic tissue mediated by the elevated levels of lipid peroxidation in both glyphosate- and Roundup-treated rats. Furthermore, increased nitric oxide (NO) and tumor necrosis factor α (TNF-α) levels were observed in the same exposed animals, suggesting a role for Roundup and its active ingredient, glyphosate, as antioxidant disruptors. Roundup and glyphosate were also capable of altering kidney function promoting an increase in blood urea [35]. Accordingly, renal and liver dysfunction were observed by Mensage et al. [36] who carried out a two-year study by giving rats water containing Roundup at a concentration of 0.1 ppb with a corresponding amount of glyphosate equal to 0.05 μg/L. Their results showed that Roundup caused an increased incidence of anatomical signs of disease and changes in blood and urine parameters that are symptomatic of functional liver and kidney failure. This investigation was also deepened through a molecular approach by analyzing the gene expression profiles in the liver and kidney. Altered expression patterns typical of mitochondrial dysfunctions and pathologies, such as fibrosis, necrosis and ischemia, were observed [35]. Another in-vivo study showed the effects of Roundup on adipose tissue and the liver, which are the main organs having a role in maintaining homeostatic energy. Adult male rats were exposed to increasing concentrations of Roundup (from 5 to 250 mg/kg body weight, [bw]) orally every day for 14 days. After 2 weeks of treatment, significant increased levels of inflammatory markers, such us interleukin (IL)-1β, TNF-α, and IL-6, were found at 100 and 250 mg/kg bw/day, together with liver damage ascribable to steatosis and non-alcoholic fatty liver. The formation of micro and macro-vesicular steatosis was observed after the treatment with the same doses of Roundup [37,38]. These doses exceed both the acceptable operator exposure level (AOEL, 0.1 mg/kg bw/day) and the acceptable daily intake (ADI, 0.5 mg/kg bw/day) for consumers fixed by EFSA [41]. However, this does not exclude that those similar alterations could be achieved with a low-dose chronic exposure to Roundup and the authors do not provide any evidence for the effects of pure glyphosate. Of note, experimental evidence from mouse models, collected from a farm, that there inhaled air samples after spraying with glyphosate, pointed out that this pesticide could represent a risk factor for the onset of occupational asthma. In fact, the exposure to an average of 17.33 µg of glyphosate-rich air samples (corresponding to about an airborne concentration of 22.59 ng/m^3^) induced an increase in cell counts of eosinophils and neutrophils and mast cells in the lungs of exposed mice. The onset of the inflammatory process was also confirmed by analyzing the cytokines pattern typically produced in the case of asthma, such as IL-5, IL-10, IL-13 and Interferon-γ (IFN-γ). In this case, the authors do not specify if farmers were exposed to pure glyphosate or GBHs, however, these results suggest that exposure to glyphosate induces damage to the epithelial barrier of the airways, the first source of contact with inhaled pesticides. The damage of the epithelium involves the release, especially, of the cytokine IL-33 and thymic stromal lymphopoietin (TSLP), which are known to amplify the inflammatory response, inducing bronchial hyperactivity [39,40].

### 3.2. Glyphosate—Induced Effects in the Intestine

Glyphosate appears to be related to intestinal inflammation [42]. The intestine, in fact, is very susceptible to the presence of pesticides, representing one of the first barriers with which exogenous substances come into contact [43]. Some studies have reported that pesticides can alter the integrity of the intestinal barrier and oxidative stress is one of the mechanisms by which these substances induce toxicity [44,45]. In line with these observations, the administration of glyphosate (at doses between 10 and 40 mg/kg) in pigs weaned for 35 days caused an increase of the enzymes superoxide dismutase (SOD), glutathione (GSH) peroxidase 1 (GSH-Px1), catalase (CAT) and levels of malondialdehyde (MDA, indicator of lipid peroxidation) in the duodenum. Increases in nuclear factor erythroid 2–related factor 2 (*Nfr2*) mRNA levels in both the duodenum and the jejunum were also found. The *Nfr2* gene encodes the major regulator of the cytoprotective response to endogenous and exogenous stress caused by ROS. In fact, under normal conditions, Nrf2 protein binds with small MAF proteins (sMAF) to the antioxidant response element (ARE) in the regulatory regions of the target genes. Thus, in the duodenum and jejunum, glyphosate-induced *Nrf2* expression, together with the reduction of the *GPx1* mRNA and the simultaneous induction of the *NF-kB* transcription factor mRNA, strongly suggests a decrease in intestinal antioxidant capacity, which can lead to chronic inflammation and tissue damage [42].Glyphosate also interferes indirectly with the intestinal immune system by creating an imbalance in resident microbiota. As previously explained, the shikimate pathway is absent in vertebrates but exists in plants and microorganisms, including gut microbiota. Glyphosate (75–300 mg/L) and the GBHs Chlorpyrifos (CPF, 50–200 µM) can influence the activity of some intestinal microorganisms such as *Escherichia coli* and *Lactobacillus reuteris*, which, in turn interact with the immune system. Indeed, these bacterial strains, if treated with glyphosate or CPF and then cultured with mucosal-associated invariant T cells, stimulate the release of pro-inflammatory cytokines such as TNF-α and INF-γ [46].

### 3.3. Glyphosate—Induced Effects in Blood Cells

It is noteworthy that our immune system is essential for survival, offering us protection against pathogens through innate immunity, the first line of defense, and the more powerful acquired immunity that develops later. The immune system, however, is a double-edged weapon. Damage to the latter can favor the onset of various diseases by causing a weak or excessive response [47].

Immunodeficiency can be primary, but it can become secondary following the exposure to chemical compounds. Growing studies have shown there is a correlation between alterations in the immune system and exposure to organophosphate pesticides like glyphosate [48,49]. Lioi et al. [50,51] were the first to report that low concentrations of pure glyphosate (from 17 to 170 µM) can induce oxidative stress in human and animal lymphocyte cultures, inducing the activity of glucose 6-phosphate dehydrogenase (G6PD), the key enzyme of the pentose phosphate pathway that ensures the NADPH necessary to preserve the intracellular pool of reduced GSH [50,51]. More recent studies conducted by Pereira et al. [52] investigated the immunological profile of a group of farmers exposed for 15 days to the use of multiple pesticides. The exposed group was made of 43 people (from 29 to 72 years old) and compared with a control group of 30 people (from 32 to 66 years old). A significant increase in monocytes, dendritic cells and total T cells was observed in the glyphosate-exposed group compared to the controls. The altered number of leukocytes was also associated to an increase in the production of the inflammatory cytokine IL-6 in exposed farmers [52]. In another in-vitro study, performed by Barbasz et al. [53], the potential toxic effect of three pesticides, including glyphosate, was tested on two human immune-cell lines: the human histiocytic lymphoma cell line (U-937) and the human promyelocytic cell line (HL-60). The concentration of pesticide chosen to treat cell lines was equivalent to the amount to which farmers or the general population are usually exposed. From a cell-vitality analysis, it was shown that cells, treated for 24 h with 3600 μg/mL of glyphosate, survived only in 20% of cases as compared with controls, thus corroborating previous results on the cytotoxicity of this pesticide. Lipid peroxidation was also assessed by determining the MDA concentration: the MDA concentration in the U-937 cell line increases four times more than the control group, suggesting that glyphosate cytotoxicity could be mediated by the induction of strong oxidative stress [53].

### 3.4. Neurodegenerative Glyphosate—Induced Effects

Environmental stressors such as pesticides can contribute to neurological disorders through mechanisms involving inflammation, oxidative stress and apoptosis; thus, long-term glyphosate exposure could cause neurodegenerative diseases. From this point of view, Cattani et al. [54] found that rat maternal sub-chronic exposure to GBHs containing 0.36% of glyphosate in drinking water and corresponding to 70 mg of glyphosate/kg bw/day affected cholinergic and glutamatergic neurotransmission in offspring's hippocampus, from both immature and adult rats. The observed decrease of glutamate uptake and increased Ca^2+^ influx, in both 15-day old and 60-day old rats, indicated a persistent glutamate excitotoxicity from the developmental period to adulthood. These events culminated in oxidative stress phenomena, astrocyte dysfunction and depressive-like behaviors [31,54].

In another in-vitro study, performed in the human neuroblastoma cell line SH-SY5Y, glyphosate (5 mM) and AMPA (10 mM) treatments induced cytotoxic effects increasing MDA levels, NO and ROS production, as well as caspase (CASP) 3/7 activity. Also found was the enhanced expression of pro-apoptotic genes such as *CASP3*, *CASP9*, *TNFα*, *Tumor Protein 53* (*TP53*), up-regulation of the Wnt pathway and down-regulation of *Growth Associated Protein 43* (*GAP43*) and *Tubulin Beta 3 Class III* (*TUBB3*) mRNA, hallmarks of neuronal development. Furthermore, the authors showed alterations in the expression profiles of several genes of cell death pathways, suggesting that glyphosate and AMPA can affect neuronal development by inducing oxidative stress and cell death via apoptotic, autophagic and necrotic pathways [34]. Although insufficient, these preliminary data on the neurotoxicity of glyphosate indicate that environmental exposure to this pesticide and its formulations could be a concern during neuronal cell development and migration. On the other hand, from the observed involvement of the Wnt pathway in the glyphosate-induced effects arises the problem that this pesticide can impair not only the normal functionality of the typical inflammation pathways but also those that link inflammation to cancer risk.

## 4. Carcinogenic and Mutagenic Effects of Glyphosate

The biotransformation of xenobiotics leads to the cellular production of reactive intermediates, such as ROS, that can damage DNA, inducing various mutations and uncontrolled proliferation that ultimately lead to cancer. In recent years, the potential genotoxic effects of glyphosate have been subject of debate both in the scientific community and among international agencies. The discrepancies between the results on glyphosate-induced DNA damage are mainly due to the different experimental methodologies used, making it difficult to reach unambiguous and clear conclusions [11].

Although the clastogenic effects of glyphosate have not been detected in bovine peripheral lymphocytes in vitro [55], other studies indicated that this herbicide has genotoxic effects at the cellular and genomic levels in different types of cells, especially peripheral blood mononuclear cells (PBMCs) [50,51]. Andreotti et al. [13] recently conducted an epidemiological study showing that, out of 54,251 people who use pesticides, 44,932 used glyphosate and 5779 developed cancers. Authors also suggested a specific correlation with the risk of developing acute myeloid leukemia (AML) in the group exposed to the highest amount of glyphosate [13]. Since DNA damage alters numerous cellular processes, evaluating the genotoxic potential of these xenobiotics appears to be crucial in assessing human health risk. The most frequent lesions that occur in DNA consist of single-strand breaks, double-strand breaks, different types of chromosomal aberrations and DNA oxidative damage involving a modification of the nitrogenous bases; however, changes in the methylation profile of several genes must be also considered as they are associated with an increased risk in developing cancer [56,57]. From this point of view, Santovito et al. [58] evaluated, in vitro, the effects of glyphosate concentrations corresponding to the acceptable daily intake (ADI) established by EFSA (0.5 μg/mL) and its submultiples on human lymphocytes. They observed that glyphosate was able to induce micronuclei (MNi) and chromosomal aberrations, such as chromatid and chromosome breaks, dicentric chromosomes, ring and acentric fragments, suggesting a cancer risk for exposed subjects [58]. These data corroborated the results previously obtained by Lioi et al. [50,51], who showed a weak but significant genotoxic effect, in terms of chromosomal aberrations and sister chromatid exchanges (SCEs)/cell, in human and bovine lymphocytes exposed to glyphosate [50,51]. The finding by Lioi et al. [50,51], indicating an increase of SCEs/cells, also suggested a reduced efficiency of DNA repair enzymes, most of which are epigenetically regulated. In another study by Wozniak et al. [57] it was determined the genotoxic potential not only of glyphosate but also of its formulation Roundup and its metabolite AMPA. It was found that all compounds can cause single-strand breaks, while glyphosate (at a concentration of 1000 μM) and Roundup at a concentration of 10 μM caused comparable DNA damage in the Comet assay. Of note, the authors suggested that DNA lesions were caused by oxidative stress, leading to the formation of 8-oxodG, which is known to favor the incorporation of adenine in place of cytosine causing G: C -> T: A transversion. They also demonstrated a greater toxicity of Roundup compared with glyphosate alone [59].

It is well known that xenobiotic substances can also induce epigenetic mutations. Changes in the level of general methylation or within the promoter regions of genes involved in different cellular processes can have important consequences for eukaryotic cells, including the increase of cancer risk [60,61,62]. From this perspective, recent studies showed that glyphosate at low concentrations (from 0.5 to 0.1 mM) reduced the methylation of *P21* and *TP53* suppressor gene promoters, which are notoriously involved in apoptotic pathways [62]. According to these findings, a global condition of DNA hypomethylation was found in PBMCs treated with higher concentrations of glyphosate (0.5 to 10 mM); however, hypermethylation was observed in the promoter region of the *p53* gene following the treatment with 0.25- and 0.5-mM [63]. Altogether these observations strongly suggest that glyphosate can induce epigenetic effects by disturbing the normal methylation processes and gene expression in human PBMCs, probably leading to cell transformation. Accordingly, the IARC working group has concluded that there is a link between glyphosate exposure and non-Hodgkin’s lymphoma [5,10,64].

The genotoxic activity of glyphosate and GBHs have also been demonstrated in the human liver-cell line HepG2. In these cells, the treatment with glyphosate and four different formulations of Roundup containing increasing amounts of the active ingredient (from 7.2 to 450 g/L) induced DNA damage and anti-estrogenic activities on estrogen receptors α and β (ERα, ERβ). Interestingly, the effects were more dependent on the formulations than on glyphosate concentration, suggesting that GBHs can be more toxic than the pure glyphosate [65]. Similar results were obtained by Koller et al. [66], who investigated the potential adverse effect that glyphosate and Roundup can cause on a human-derived buccal epithelial cell line, TR146. In this case, the comet assay and the MNi tests demonstrated that the substances at the concentration of 20 mg/L promote DNA damage, probably by inducing single-strand breaks, apurinic sites, MNi and nuclear buds (NB) and, in this study, Roundup was more active than glyphosate [66]. Furthermore, in Hep-2 cells exposed to glyphosate at a concentration range of 3 to 7.5 mM for 4 h, the comet assay revealed a statistically significant increase in DNA damage [67]. In addition to the evident genotoxic effect that glyphosate and GBHs can have, it has been seen that, in some forms of cancer, they can favor the maintenance of tumor condition by inducing multidrug resistance (MDR). Despite originating in healthy human cells, cancer cells undergo rapid mutations and develop different mechanisms of adaptation to stress conditions that can ensure greater survival. These include the MDR mechanism. The acquisition of the MDR phenotype is one of the main problems encountered in the treatment of various forms of cancer such as glioblastoma multiforme (GBM). This phenomenon is related to an overexpression of ABC membrane transporters (such as P-glycoprotein) [68], breast cancer-resistance protein (Bcrp) and glutathione s transferase (GSTs) [69]. The chronic presence of pesticides in the body could activate MDR mechanisms. In the human glioblastoma cell line U87 it has been shown that, consequent to exposure to the combined action of various pesticides (including glyphosate), cells develop resistance to chemotherapeutic agents. In these resistant U87 cells, there was an increase in the expression of all biomarkers involved in the MDR mechanism (such as GST, P-gp/ABC, MRP) and a greater resistance to apoptosis and oxidative stress [70]. From this point of view, it would be necessary to increase our knowledge on the adverse effects of the contemporary exposure to several pesticides, as this could occur, particularly, for farmers.

Other studies on the human cancer cell lines HEC1A (endometrial cancer cell line) and MDA-MB-231 (estrogen receptor (ER) negative breast cancer line) the genotoxic effects of glyphosate and its co-genres Roundup and Wipeout were also correlated with the estrogen receptors status. These effects were found mainly by using short time treatments and moderate doses (from 75 to 500 µg/mL); glyphosate induced significant DNA damage in both cell lines. Indeed, the toxic effect was higher in HEC1A (ER positive) compared with MDA-MB-231 (ER negative). MDA-MB-231 are considered hormone-independent cells that express low levels of the ERα and ERβ receptors and, therefore, in this case it is possible that glyphosate acts through a non-estrogenic mechanism, suggesting a potential endocrine-disruptive role for this pesticide [71]. In line with this hypothesis, in T47D cells, a human hormone-dependent breast-cancer cell line, glyphosate concentrations ranging from 10^−12^ to 10^−6^ M has been shown to have a pro-proliferative effect. The estrogen response element (ERE) luciferase assay—performed by using the T47D-KBluc cell line transfected with a triplet ERE-promoter luciferase reporter-gene construct—gave evidence that glyphosate behaves like a xenoestrogen that can induce ERE activation. This mechanism is inhibited by the estrogen antagonist ICI 182,780 corroborating that glyphosate activity is mediated by ERs. Therefore, although the binding of glyphosate to ERs is not yet documented, its ability to stimulate the ERE-regulated transcription suggest that it could have a stimulatory effect through an ER-dependent mechanism [72]. These last observations pointed out that, in addition to cytotoxic and genotoxic effects on various experimental model systems, glyphosate also interferes with the estrogen pathway in a manner that remain to be elucidated. Moreover, as summarized in the next paragraph, GBHs have been reported to induce adverse effects in animal reproduction, including disruption of key regulatory enzymes in androgen synthesis and alteration of serum levels of estrogen and testosterone [73].

## 5. Effects of Glyphosate on Reproduction and Development

**Figure 4:**
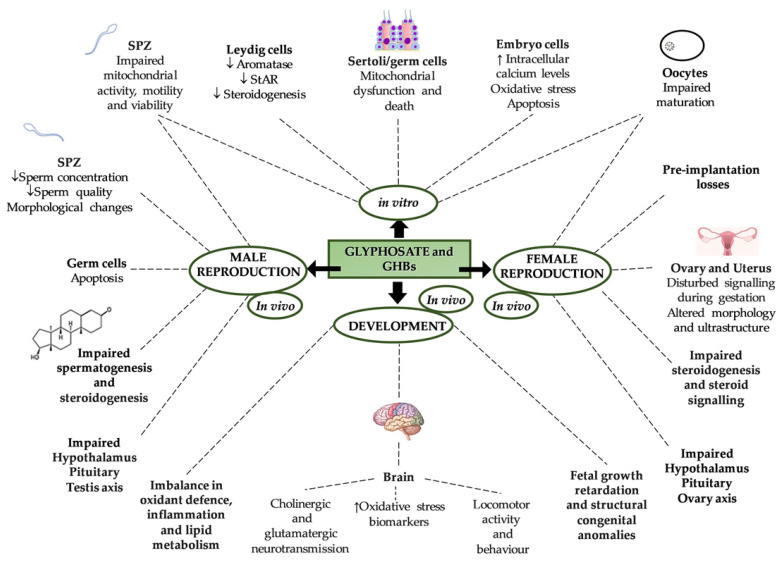
Schematic representation of glyphosate’s and glyphosate-based herbicide’s (GBHs) -induced effects on mammalian reproduction, fertility and development. ↓: decreased; ↑: increased.

The production of high-quality gametes is the first step for successful reproduction and species conservation. Notably reproduction is under the control of the hypothalamus–pituitary–gonadal (HPG) axis and finds the main actors in the hypothalamic gonadotropin releasing hormone (GnRH), pituitary gonadotropins and sex steroids [74]. Many environmental factors, from nutritional cues, drug abuse, stressors or environmental pollutants, among others, affect reproduction at multiple levels along the HPG axis, with consequences for gamete quality, successful reproduction, embryo development and offspring health, as previously reviewed [75,76,77,78,79]. In this respect, in spite of the relative safety of glyphosate, in-vivo and in-vitro studies involving different organisms and cell types reported various adverse effects on reproduction (Figure 4), but several controversies concern chemical composition (glyphosate alone or in formulation), doses and exposure windows.

Therefore, Table 1 summarizes the significant outcomes in mammalian reproduction following in-vivo and in-vitro treatments of glyphosate and GBHs, alone or in combinations. In general, glyphosate or GBHs exposure during neonatal, (peri)pubertal or adult life interferes in the physiology of the HPG axis affecting the hormonal milieu critical for reproduction, sex-steroids production and signaling pathways (details and references in Table 1).

Usually, formulants have more deleterious effects than glyphosate alone because of possible cumulative effects on endocrine and reproductive endpoints [106]. As summarized in Table 1, in female reproduction, glyphosate and GBHs exposure has consequences for post-natal ovarian and uterine development, puberty onset, oocyte maturation, sperm-oocyte binding ability and early embryo development, implantation and successful pregnancy. Consistently, germ-cell loss, impaired spermatogenesis and negative effects on sperm quality have been reported in males. In addition, toxicity on both nurse Sertoli cells and steroid-secreting Leydig cells have been observed [98,99]. In this respect, particular interest is deserved for the direct inhibitory effect of glyphosate and its formulations on the activity of P450scc Aromatase (in embryonic human cells), placental cell lines and tumor MA-10 Leydig cells [98,103,104] and the widespread interference in sex-steroid signaling, are observations that corroborate the hypothesis that glyphosate and GBHs need inclusion in the list of endocrine-disrupting chemicals (EDCs). Imbalance in the oxidative stress response is a common feature of glyphosate and GBHs in both sexes, but there is the need for additional studies to fully elucidate the molecular mechanisms and the possible adverse effects on human reproduction. Worthy of note, the aforementioned consequences of glyphosate or GBH exposure on reproduction have been largely analyzed in cell lines and rodents using different doses and formulations, but data in human and domestic animals are very limited. Anifandis and coworkers, in 2017 and 2019 [101,102], analyzed the direct effects of Roundup and glyphosate on sperm motility and DNA fragmentation in human sperm collected from n = 66 and n = 30 healthy volunteers, respectively; their studies, for the first time, revealed that the tested herbicides significantly reduced sperm motility without any effects on DNA integrity [101]. Consistently, in 2020 Nerozzi and coworkers [100] confirmed these glyphosate and Roundup effects on sperm motility using fresh commercial pig semen doses, whereas, in the same year Cai and coworkers [105] reported the deleterious effects of Roundup on bovine preimplantation embryos [105]. Recently the ability of glyphosate and GBHs to induce epigenetic changes emerged in cell lines and rodents [107], for recent review; but, this point has yet to be unraveled in gametes. Considering that gamete epimutations may interfere in early embryo development and health, once again there is the need of further studies on the reproductive effects of glyphosate and GBHs in order to elucidate the related molecular mechanisms and to preserve gamete quality, reproduction health and offspring health.

Glyphosate accumulates in bird eggs [108] and causes developmental and reproductive alteration in nematodes, fish and amphibians, among others [109,110,111,112,113,114,115] and it influences antioxidant defenses, reproduction and microbioma in avian models [116]. Equally warring are data concerning the chronic exposure, in utero, regarding lactation and weaning in mammalian animal models (Table 2).

Apart from the effects on maternal brain plasticity, licking behavior and the microbiome [133], exposed offspring exhibit several long-term alterations in brain, mammary gland, inflammation and lipid metabolism, oxidative stress response and reproduction (details and references in Table 2). Epigenetic changes in DNA methylation status, histone modifications and the production of non-coding RNA, such as microRNA and circular RNA, have also been reported in specific brain areas, as recently reviewed [107].

Focusing on the reproductive phenotype of the exposed offspring, glyphosate and GBHs may program the fetus to induce reproductive damage in adulthood. In fact, impairment of the HPG axis, sex-steroid production and sex-steroid signaling, altered uterine physiology (possibly associated with implantation failures), decreased spermatogenesis and sperm quality are the main outcomes in F1 offspring. Interestingly, the modulation of the epigenetic machinery has been reported in fish [134], and epigenetic mechanisms involving *ESR1*, the gene encoding for ERα, have been reported in F1 female offspring in rats [124]. Contrarily, Milesi 2018 et al. [121] suggested that in mammals the perinatal exposure to low doses of glyphosate formulation impaired female reproductive performance and induced fetal growth retardation and structural congenital anomalies in F2 offspring [121].

As for in-vitro and in-vivo exposure at neonatal and peripubertal phases and in adult life, the aforementioned effects are stronger in exposure to glyphosate formulations than in glyphosate alone.

In mammals, glyphosate and GBHs effects seems to be stronger in female reproduction than males, though androgenic effects have been reported. In this respect, the anogenital distance (AGD) is considered an early-life biomarker of fetal androgen exposure in several species, thus representing a reproductive toxicity endpoint to evaluate chemicals in animal studies [135]. Furthermore, multiple epidemiological studies have shown that AGD measurements in infants are sensitive to in-utero exposures to EDCs [136]. Gestational exposure at doses of glyphosate considered to be “safe” for human health significantly increased AGD in male and female rat pups, with Roundup treatment capable of delaying the age of first estrous and a parallel increase in serum testosterone in the adults [123].

Recently, these possible androgenic effects were investigated in humans in a pilot study enrolling 94 pregnant women. In their 2021 study, Lesseur and coworkers [137] measured glyphosate and its degradation product, AMPA, in second trimester maternal urine samples by ultra-high-performance liquid chromatography-tandem mass spectrometry, revealing glyphosate and AMPA presence in 95% and 93% of the samples (median 0.22 ng/mL and 0.14 ng/mL), respectively. Then, urinary glyphosate levels in the mothers were correlated to the anogenital distance (AGD) in female and male infants (n = 45 and 49, respectively). While no correlation was observed in male infants, increased AMPA was associated with longer anofourchette distances in female infants. Although preliminary, data in humans might suggest the possible sex-specific effects of glyphosate [137], confirming the androgenic effects of Roundup previously reported in rats [123].

Taking the above together, a possible health risk exists; larger studies should evaluate the possible developmental and reproductive effects of glyphosate.

## 6. Conclusions

**Figure 5:**
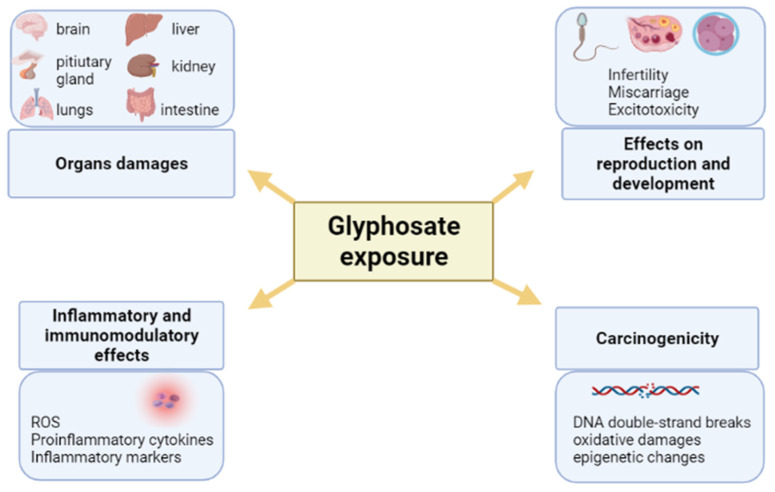
Schematic overview of glyphosate- and GBHs-induced effects. Image has been created with BioRender.com, accessed on 1 November 2021.

Exposure to pesticides is known to cause irreversible damage to the environment and serious consequences for human health. Scientific evidence has shown that exposure to glyphosate and GBHs can predispose humans to the onset of systemic inflammatory diseases, cancer and neurological disorders. However, the molecular mechanisms responsible for the observed effects are not fully understood. The similarity between glyphosate and its metabolite, AMPA, to glycine and glutamate could only explain in part some observed neurotoxic and cytotoxic effects. On the other hand, the recent findings on glyphosate action as an endocrine disruptor could account for its capacity to induce, among others, hormonal imbalances with adverse effects on fertility and reproduction (Figure 5). As for in-vitro and in-vivo exposure at the neonatal peripubertal phases, and in adult life, it appears that all glyphosate- and GBHs-induced effects are stronger in formulations thereof than from glyphosate alone. In mammals, glyphosate and GBHs effects seems to be stronger in female reproduction than males.

Indeed, from the overall data, a number of criticisms arise: (i) studies carried out in vivo and in vitro do not give final indications of the acceptable daily intake (ADI); (ii) the existing data about glyphosate genotoxicity and cytotoxicity are still conflicting, as a result of different experimental conditions used in the research thereabout; (iii) there are no data about glyphosate-induced long-term effects on general populations or exposed farmers; (iv) GBHs seem to exhibit higher toxic effects than glyphosate alone, but studies on this matter are still few. Therefore, to date, it is not possible to have a univocal opinion on the safety of glyphosate and it appears that the human health risk associated with glyphosate could still be underestimated. The IARC has included glyphosate into the group 2A, “probably carcinogenic to humans”; while the EFSA has conducted a technical assessment, according to which glyphosate does not constitute a carcinogenic hazard for human health. The discrepancy between IARC and EFSA classification is ascribable mainly to the diverging views between the two groups of experts [41]. This is because, on the one hand, the IARC analyzed both glyphosate and GBHs toxicity studies, while EFSA analyzed only those on glyphosate. On the other, the number of epidemiological studies included in the IARC monograph are fewer than those evaluated by EFSA. Moreover, the IARC considered as reliable the carcinogenic effects and genotoxicity, oxidative stress and DNA damage obtained in-vivo from laboratory animals and in vitro, while EFSA, even while recognizing the importance of these studies, has concluded that there is limited epidemiological evidence for a correlation between glyphosate exposure and cancer [10,41]. Currently, the approval period for the use of glyphosate in the EU extends until 15 December 2022. In the meantime, it would be desirable to investigate the possible role of glyphosate and GBHs exposure on the onset of neurodegenerative and behavior disorders. Moreover, it could be useful to perform studies leading to a deeper knowledge of the possible involvement of glyphosate and its formulations in the pathogenesis and/or development of human cancer. Since glyphosate has been found in maternal milk, other aspects should be investigated, such as the pharmacokinetics of the pesticide and its ability to induce epigenetic transgenerational inheritance, in order to better establish the real exposure limits preserving human health and especially that of future generations.

## Figures and Tables

**Table 1:** In-vivo and in-vitro administration of glyphosate and glyphosate-based herbicides (GBHs): effects on reproduction in mammals.

Experiment	Species/Cell Types	Treatment	Effects	Reference
In vivo	Newborn female rats	Subcutaneous injection: 2 mg/kg/day GBH (66% glyphosate in potassium salt) on PND1, 3, 5 and 7	↑Number of resorption sites on GD19, associated with altered decidualization response Morphological changes at the implantation site ↓Estrogen and progesterone receptors (ER and PR) ↓*COUP-TFII* (*Nr2f2*) and *Bmp2* mRNA ↑HOXA10 and Ki67	[80]
In vivo	Newborn female rats	Subcutaneous injection: 2 mg/kg/day GBHs on PND1 to 7	Disturbed uterine signaling (*Wnt5a*, *β-catenin*, *Wnt7a*, *Dkk1* and *sFRP4*) during gestation	[81]
In vivo	Newborn female rats	Subcutaneous injection: 2 mg/kg/day GBH on PND1 to 7	↑LE hyperplasia ↑Stromal and myometrial thickness ↑Proliferation and endometrial hyperplasia Altered expression of proteins involved in uterine organogenetic differentiation (i.e., PR and Hoxa10, and ERα)	[82]
In vivo	Female Wistar rats (pups)	Subcutaneous injection: Endosulfan (600 μg/kg bw/day), GBHs (2 mg/kg b.w/day) or a mixture (mix) from PND1 to 7	*GBHs and mix:* ↑Incidence of luminal epithelial hyperplasia ↑*PR* and *Hoxa10* expression ↑Post-implantation losses during adulthood *Endosulfan:* Modified *ERα* and *Hoxa10* expression. ↑Pre-implantation losses	[83]
In vivo	Female weaned piglets	Glyphosate concentrations 10, 20, and 40 mg/kg into the feed	No significant effect on vulvar size and reproductive organs Altered tissue morphology and ultrastructure in uterus and ovary ↑Oxidative stress in uterus ↑LHRH/GnRH ↑Testosterone ↓FSH	[84]
In vivo	Prepubertal female ewe lambs	Oral and subcutaneous exposure to a GBHs (2 mg/kg/day) from PND1 to PND14	*PND45*: Altered follicular dynamics ↑Proliferation of granulosa and theca cells ↓*FSHR* and *GDF9* mRNA ↓Proliferation in the uterus	[85]
In vivo	Female Friesian ewe lambs	GBHs (2 mg/kg/day) through subcutaneous. injections from PND1 to PND14	*PND45* (uterus): ↓Cell proliferation ↑p27 ↑Insulin-like growth factor binding protein 3 ↓ERα in the LE and GE and in the SS ↓PR expression in the LE ↑PR in the GE and SS ↓Gene expression in the uterus (i.e., *Wnt5a* in the GE, *Wnt7a* in the SS, *β-catenin* in the LE and GE, *Hoxa10* in the SS, and *Foxa2* in the GE)	[86]
In vivo	8-weeks-old male Kunming mice	Gavage: Roundup, 60, 180, 540 mg/kg	Impaired spermatogenesis, ↓Sperm motility and concentration ↑Sperm deformity rate ↑Apoptosis of germ cells with mechanism involving the over-expression of the *X-linked inhibitor of apoptosis-associated factor 1* (*XAF1*)	[87]
In vivo	Prepubertal male Wistar rats	Oral gavage: 5, 50 or 250 mg/kg bw glyphosate-Roundup Transorb from PND23 to PND53	Dose dependent changes in spermatogenesis progression ↓Seminiferous epithelium height ↓Serum levels of testosterone	[88]
In vivo	4 weeks-old male Sprague-Dawley rats	Oral gavage: two weeks exposure to either glyphosate (2.5 and 25 mg/kg bw/day) or herbicide formulation Glyfonova	*Glyfonova*: Slight increase in the expression of the steroidogenic genes *Cyp11a1* and *Cyp17a1*	[89]
In vivo	Male Wistar rats (12 weeks old)	Dietary administration: 375 mg/kg/day glyphosate ± 20 mg/kg/day resveratrol	↓Sperm motility ↓Sperm plasma membrane integrity ↓Glutathione level ↓Superoxide dismutase ↑Abnormal sperm rate ↑Malondialdehyde level ↑DNA damage All the effects were reversed by resveratrol co-administration	[90]
In vivo	Male Sprague Dawley rats	Glyphosate 5, 50, 500 mg/kg by gavage	↓Average daily feed intake at dose of (50 mg/kg dose) ↓Weight of seminal vesicle gland and coagulating gland (500 mg/kg dose). ↓Total sperm count (500 mg/kg dose) No effects on testosterone, estradiol, progesterone and oxidative stress parameters	[91]
In vivo	Sexually mature male guinea pigs	Oral exposure: Willosate 186, 280 and 560 mg/kg daily for 60 days	↓Sperm motility, viability and concentration ↑Sperm morphological alterations	[92]
In vitro	Mouse Oocytes	500 μM Glyphosate	↓Germinal vesicle breakdown and first polar body extrusion Abnormal spindle morphology and DNA double-strand breaks ↑Oxydative stress ↑Mitochondria aggregation ↓Mitochondria membrane potential ↓Expression levels of autophagy-related genes (*lc3*, *atg14*, *mTor*) and proteins (LC3, Atg12)	[93]
In vitro and in vivo	Mouse oocytes	In vitro: 0.00001%, 0.00005%, or 0.00025% GBHs ± melatonin (10 and 100 μM) In vivo: GBHs (0.0005% Roundup solution) daily administered in drinking water for 21 days ± melatonin (0, 0.15, and 1.5 mg/kg bw), once a day through intragastric administration	Impaired oocytes meiotic maturation ↓First polar body extrusion, disorganized spindle morphology, misaligned chromosomes, and ROS production ↑Apoptosis rate ↓Sperm-binding ability and disrupted early embryo cleavage GBHs effects were reversed by in vitro/in vivo melatonin treatment with mechanisms involving the membrane GPER	[94]
In vitro	Pig oocytes	0, 5, 10, 100, 200 and 360 µg/mL Glyphosate or Roundup at the same glyphosate -equivalent doses	*Glyphosate:* No effect on nuclear maturation and embryo cleavage, impaired oocyte developmental competence in terms of blastocyst rate and cellularity *Roundup*: more toxic than pure glyphosate, altered steroidogenesis ↑ROS levels	[95]
In vitro	Rat isolated testicular cells and co-colture of germ cells-Sertoli cells	Glyphosate and Roundup: 1–10,000 ppm, from 1 to 48 h	*Leydig cells*: damaged (Roundup, 1–48 h) *Sertoli cells*: toxic effect (glyphosate alone) *Germ cells*: necrosis (Roundup, 24–48 h) and apoptosis (high doses) *Co-colture assay*: apoptosis of Sertoli cells and germ cells (at high doses) ↓Testosterone levels (Roundup and glyphosate 1 ppm)	[96]
In vitro	Sertoli cells from PND30 (prepubertal) Wistar rats	Acute Roundup exposure at low doses (36 ppm or 0.036 g/L) for 30 min	Endoplasmic reticulum stress Depletion of antioxidant defences Cell death	[97]
In vitro	Immature Sertoli cell line (TM4)	Commercially availableGBHs: Genamin T200 (732 g/L Polyethoxylated tallowamine, 60–80% POE (15) tallowamine (POE-15)); Glyphogan (360 g/L of glyphosate); Roundup Bioforce (360 g/L of glyphosate)	Mitochondrial dysfunction Disruption of cell detoxification systems Lipid droplet accumulation Mortality at sub-agricultural doses Formulants have more deleterious effects than glyphosate	[98]
In vitro	MA-10 Leydig cells	Roundup (180 g/L glyphosate)	Inhibition of dibutyryl [(Bu)(2)]cAMP-stimulated progesterone production ↓Activity of Aromatase No effect on the activitiy of 3β-HSD ↓Steroidogenesis by disrupting StAR protein expression	[99]
In vitro	Pig semen	0–360 µg/mL glyphosate or Roundup	*Glyphosate*: ↓Sperm motility, viability, mitochondrial activity ↓Acrosome integrity *Roundup*: ↓Sperm motility (≥5 µg/mL glyphosate-equivalent concentration) ↓Mitochondrial activity (25 µg/mL glyphosate-equivalent concentration) ↓Sperm viability and acrosome integrity (≥100 µg/mL glyphosate-equivalent concentration)	[100]
In vitro	Human sperm (n = 66 healthy men)	1 mg/L Roundup	↓Sperm motility and mitochondrial dysfunction	[101]
In vitro	Human sperm (n = 30 healthy men)	0.36 mg/L glyphosate	↓Sperm progressive motility (1 h post-treatment)	[102]
In vitro	Human cell lines: (JEG3 placental cell lines, HUVEC primary neonate umbilical cord vein, and 293 embryonic kidney HEK293)	GBHs in Roundup formulations	Cell death within 24 h in all cell lines	[18]
In vitro	Human cell lines (JEG3 placental cell lines and HEK293)	Glyphosate alone and in 14 ot its formulations	Toxic effects ↓Ativity of Aromatase	[103]
In vitro	Human JEG3 placental cell lines	0.05–2% glyphosate and Roundup (360 g/L glyphosate)	Toxic effects with concentrations lower than those found with agricultural use ↓Aromatase activity	[104]
In vitro	Bovine preimplantation embryos	Roundup 0.01~2% (36~7200 ppm, containing 36~7200 mg/L glyphosate)	0.01~2% Roundup doses are toxic to bovine embryos	[105]
In vitro	Bovine preimplantation embryos	Roundup 0, 0.45, 0.9, and 1.8 ppm	↑Intracellular calcium levels (2-cells embryo) ↑Oxidative stress (2-cells embryos) ↑Apoptosis (bovine blastocysts)	[105]

AR, androgen receptor; bw, body weight; Cyp11a1, cytochrome P450 family 11 subfamily A member1; Cyp17a1, cytochrome P450 family 17 subfamily A aember 1; ER, estrogen receptor, FSH, follicle-stimulating hormone; FSHR, follicle-stimulating hormone receptor; GBHs, glyphosate based-herbicides; GD, gestation day; GE, glandular epithelium; GnRH, gonadotropin releasing hormone; GPER, G protein-coupled estrogen receptor 1; 3β-HSD, 3β-hydroxysteroid dehydrogenase; LE, luminal epithelium; PND, post-natal day; PR, progesterone receptor; ROS, reactive oxygen species; SS, subepithelial stroma; StAR, steroidogenic acute regulatory protein; ↑, statistically significant increase; ↓, statistically significant decrease (*p* < 0.05 at least).

**Table 2:** Exposure to glyphosate and glyphosate-based herbicides (GBHs) during pregnancy and lactation: effects on the development, reproduction and fertility of the offspring.

Species	Dams’ Treatment	Exposure Route	Effects on Dams and Litter Size	Effects on F1 (Males)	Effects on F1 (Females)	Reference
Mouse	0.5% glyphosate-Roundup from GD4 all over lactation period	Drinking water	Reduced bw gain during gestation no effects on litter size	Delayed testicular descent *PND150*: ↓SPZ in cauda epididymis ↓Epithelial height within the seminiferous epithelium ↑LH in plasma ↑Intratesticular testosterone levels	NA	[117]
Mouse	0.5, 5 and 50 mg/kg/day glyphosate or Roundup 3 Plus from ED10.5 to 20 PND	Drinking water	NA	*PND20*: Altered testis morphology (glyphosate) *PND35*: ↓Serum testosterone levels (glyphosate) ↓SPZ, (0.5 mg/kg/day Roundup and 5 mg/kg/day glyphosate) ↓Undifferentiated spermatogonia (5 mg/kg/day glyphosate) *8-month-old animals*: ↓testosterone (GBHs)	NA	[118]
Mice	0.5% glyphosate from GD1 until 30 days after birth	Drinking water	NA	↑Risk of jejunum inflammation and dysfunction in adulthood when combined with a high-fat diet	NA	[119]
Wistar rats	0, 50, 150 or 450 mg/kg glyphosate during pregnancy and lactation	Drinking water	NA	*Puberty:* ↓Serum testosterone levels *Adulthood:* ↓Sperm number in epididymis tail ↓Daily sperm production ↑Abnormal sperms ↑Spermatid degeneration	Delay in Vaginal canal-opening	[120]
Wistar rat	2 mg or 200 mg of glyphosate/kg bw/day from GD9 until weaning	Food	NA	NA	*F1:* No alteration in bw gain or vaginal opening onset ↓Implantation sites *F2*: Delayed growth ↓Foetal weight and length ↑Incidence of small for gestational age foetuses ↑Placental weight and placental index structural ↑Congenital anomalies like conjoined foetuses and abnormally developed limbs	[121]
Rats	GBHs (containing 66.2% of glyphosate potassium salt) or glyphosate (2 mg/kg/day) from GD9 until weaning	Orally	NA	NA	↓Preimplantation ↑17β-oestradiol serum level ↑ERα in the uterus ↓*PR* mRNA (glyphosate) ↓Uterine implantation-related genes (i.e., *Hoxa10* and *Lif*)	[122]
Sprague Dawley rats	Glyphosate alone and Roundup Bio flow, 1.75 mg/kg bw/day from GD6 up to PND120	Drinking water	NA	*PND4:* ↑AGD (all treatments) *ADULTS:* ↑plasma TSH (glyphosate) ↓DHT (Roundup) ↑BDNF (Roundup)	*PND4*: ↑AGD (all treatments) *ADULTS:* Age at first oestrous significantly delayed (Roundup) ↑Serum testosterone (Roundup)	[123]
Rats	350 mg glyphosate/kg bw/day from GD9 until weaning	Food	NA	NA	↑*ERα*-O mRNA variant in uterus Epigenetic changes in the *Esr1-O* promoter (i.e., ↓DNA methylation ↑Histone H4 acetylation ↑Histone H3 lysine 9 trimethylation (H3K9me3) ↓H3K27me3)	[124]
Mouse	GBHs (250 or 500 mg/kg) from GD0 to PND21	Oral gavage	Impaired maternal behaviour fertility and reproduction	Global delay in innate reflexes and a deficit in motor development Hippocampal dysfunctions with behavioural and cognitive impairment		[125]
Wistar rats	0.65 or 1.30 g/L of glyphosate from GD0, until weaning (PND21)	Drinking water	NA	Neurobehavioral alterations (i.e., early onset of cliff aversion reflex and early auditory canal opening, decrease in locomotor activity and in anxiety levels)		[126]
Wistar rats	0.65 and 1.30 g/L of pure glyphosate from GD0, until weaning (PND21)	Drinking water	NA	Alterations in brain oxidative stress biomarkers and glutamatergic and cholinergic systems		[127]
Wistar rats	5 and 50 mg/kg/day Roundup, (per os) from GD18 to PND5	Drinking water	NA	Altered expression of genes associated with oxidant defence, inflammation and lipid metabolism		[128]
Wistar rats	1% GBH (0.36% glyphosate) from GD5 until PND15 or PND60	Drinking water	NA	Oxidative stress and depressive-like behaviour at PND60 Impaired cholinergic and glutamatergic neurotransmission at PND15 and PND60 Altered serum levels of the astrocytic protein S100B at PND15 and PND60		[54]
Rats	GBH (66.2% glyphosate in potassium salt) 3.5 or 350 mg/kg bw/day GD9 until weaning	Orally exposed through the food	NA	*PND21*: No differences in mammary gland development or in oestradiol and testosterone levels *PND60*, *GBHs 3.5 mg/kg/day exposed animals*: ↑AR protein expression *PND60*, *GBHs 350 mg/kg/day exposed males*: ↓Proliferation index and less developed mammary gland ↑PRL serum levels *Both exposed groups*: ↓*ESR1* expression by means of hypermethylation of *ESR1* promoter		[129]
Rats	GBH (66.2% glyphosate in potassium salt) 3.5 or 350 mg/kg bw/day from GD9 until weaning	Orally exposed through the food	NA	↓Proliferation index in GBHs 3.5-exposed animals ↓mRNA levels of *ESR1*, *Ccnd1*, *Areg*, *IGF1*, *EGFR* and *IGF1R* ↓p-Erk1/2 protein		[130]
Wistar rats	5 mg/kg/day or 50 mg/kg/day Roundup from GD18 to PND5	Oral gavage	NA	↓Deiodinases 2 (*Dio2*) and 3 (*Dio3*) and TH transporters *Slco1c1* and *Slc16a2* mRNA within the hypothalamus ↑*Dio2*, thyroid hormone receptor genes (*Thra1* and *Thrb1*), and *Slc16a2* within the pituitary. ↑*Thra1* and *Thrb1* mRNA in the liver ↑*Dio2*, *Mb*, *Myh6* and *Slc2a4* mRNA expression in the heart		[131]
Wistar rats	1% Roundup (0.38% glyphosate) from GD5 and up to lactation day 15	Drinking water	NA	Excitotoxicity and oxidative stress in rat hippocampus		[31]
Wistar rats	Pure glyphosate (24 or 35 mg/kg) every 48 h from ED8 until ED20, every 48 h	Intraperitoneal injections	NA	Dose dependent changes in reflexes development, motor activity and cognitive function, via inhibition of Wnt5a-CaMKII signalling pathway.		[132]

AGD, anogenital distance; AR, Androgen Receptor; BDNF, Brain-Derived Neurotrophic Factor; bw, body weight; DHT, Dihydrotestosterone; ED, embryonic day; EGFR, Epidermal Growth Factor Receptor; ER, Estrogen Receptor; ESR, Estrogen Receptor gene; GBHs, glyphosate based-herbicides; GD, gestation day; IGF1, Insulin Growth Factor 1; IGF1R, Insulin Growth Factor 1 Receptor; LH, Luteinizing Hormone; NA, not assayed/no information about; PND, post-natal day; PR, Progesterone Receptor; PRL, Prolactin; SPZ, spermatozoa; TH, Thyroid Hormone; TSH, Thyroid-Stimulating Hormone; ↑, statistically significant increase; ↓, statistically significant decrease (*p* < 0.05 at least).

## Data Availability

Not applicable.
